# Supplementary material for: Revisiting optical properties of MgB2 with a high-quality sample prepared by a HPCVD method
Source: Sci Rep. 2017 Aug 21;7:8977. doi: 10.1038/s41598-017-09248-4 (PMC5567084; doi:10.1038/s41598-017-09248-4)
Supplement: Supplementary file 1 — Supplementary information [file 41598_2017_9248_MOESM1_ESM.pdf]

Revisiting optical properties of MgB_2 with a high-quality sample prepared by a HPCVD method.

Yu-Seong Seo, Jae Hak Lee, Won Nam Kang, & Jungseek Hwang*

Department of Physics, Sungkyunkwan University,

Suwon, Gyeonggi-do 16419, Republic of Korea

(Dated: July 15, 2017)

Abstract

In this supporting material we describe three topics to help readers of the main text. The three topics are (I) surface contamination effect by exposure to air, (II) phonon screening effect by the charge carrier density, and (III) simulation formalism for optical constants including reflectance out of the electron-phonon spectral density, $\alpha^2F(\omega)$.

*Correspondence to [email: jungseek@skku.edu].

I. SURFACE CONTAMINATION EFFECT BY EXPOSURE TO AIR

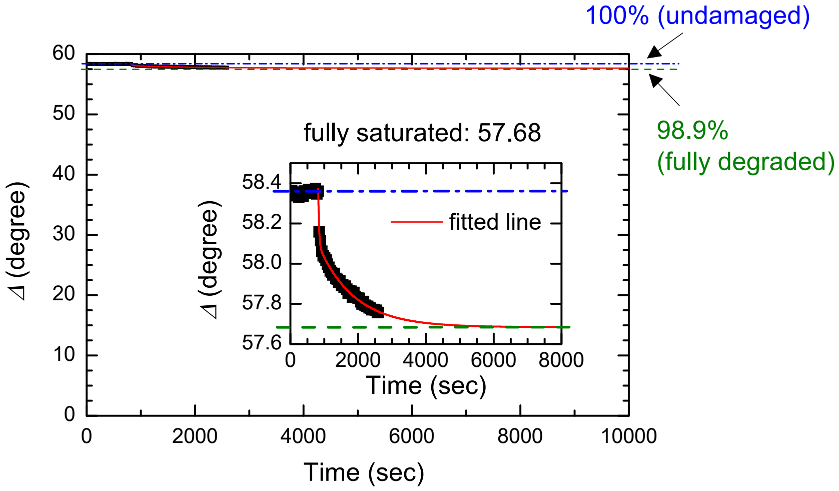

FIG. S1: The ellipsometric parameter (Δ) change as a function of the exposure time republished from Fig. 13 of a literature[S1] (http://dx.doi.org/10.1103/PhysRevB.73.104509) in a full scale of the vertical axis. In the inset we display a magnified view. The solid red line is a fit to the data with an exponential decay function and the dashed green line shows the saturated value.

There was an ellipsometry study on surface sensitivity of an MgB_2 single crystal to air[S1]. In the paper the authors showed that when the surface of their sample was exposed to air the ellipsometric parameter, Δ, was affected immediately due to the formation of a contamination layer as shown in the inset of Fig. S1. We estimate the saturated surface degradation effect using an approximate exponential decay function. The estimated saturated effect is around 1.1% of the undamaged value at ~10,000 cm^{-1} with the incident angle of 70°. This small saturated surface degradation (~1.1%) indicates that the only top layers near surface of the sample are contaminated by the exposure to air. Furthermore, if one consider the frequency-dependence of the skin depth the surface degradation effect will be even smaller in the FIR region. The skin depth (δ) is inversely proportional to the square root of the frequency (ω), *i.e.* $\delta \propto 1/\sqrt{\omega}$. The skin depth will be 10 times larger at a frequency of 100 cm^{-1} compared with the value at 10,000 cm^{-1}. Therefore, the surface effect in FIR region will be roughly 10 times smaller; then the saturated degradation will be 0.11% of the undamaged value. Furthermore, a smaller incident angle (~10°) compared to 70° may reduce the surface contamination effect further. This resulting surface degradation effect is practically too small to be measured by our optical measurement setup in the FIR region.

II. PHONON SCREENING EFFECT

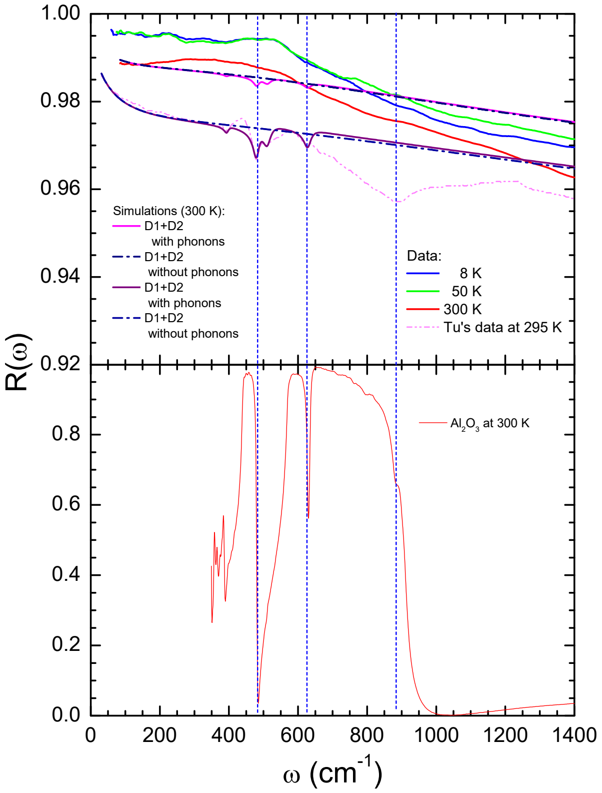

FIG. S2: (Upper frame) We display measured reflectance spectra of our MgB_2 at three temperatures (8, 50, and 300 K) and Tu *et al.*'s MgB_2 data[S2] at 295 K. We also show simulated reflectance spectra at room temperature using a simple Drude-Lorentz model. The parameters are described in detail in the text. (Lower frame) We display our measured reflectance of bare Al_2O_3 sample.

The optical phonon modes can be screened by charge carriers. Therefore, the more charge carriers can screen more strongly the phonon modes. In the upper frame of Fig. S2 we display our measured reflectance data of MgB_2 at 8, 50, and 300 K and Tu *et al.*'s data at 295 K and additional simulations at room temperature using a Drude-Lorentz model. The complex dielectric function ($\tilde{\epsilon}(\omega)$) can be written in the D-L model as

$$\tilde{\epsilon}(\omega) \equiv \epsilon_1(\omega) + i\epsilon_2(\omega) = \epsilon_H - \sum_{k=1}^{2} \frac{\omega_{p,D_k}^2}{\omega(\omega + i1/\tau_{imp,k})} + \sum_l \frac{\omega_{p,l}^2}{\omega_l^2 + \omega^2 + i\omega\gamma_l}, \tag{S1}$$

where ϵ_H is the background dielectric constant, ω_{p,D_k} and $1/\tau_{imp,k}$ is the plasma frequency and the impurity scattering rate of the kth Drude component, respectively, and $\omega_{p,l}$, ω_l,

and γ_l are, respectively, the plasma frequency, the resonant frequency, and the damping constant of the lth Lorentz component. We note that the Drude component can be used to describe free charge carriers and the Lorentz one can be used to describe the localized charges including phonon excitations. For this simple simulation we used the Lorentz modes to describe the phonon modes. The reflectance ($R(\omega)$) can be related to the dielectric function as

$$R(\omega) = \Big|\frac{\sqrt{\tilde{\epsilon}(\omega)} - 1}{\sqrt{\tilde{\epsilon}(\omega)} + 1}\Big|^2 = \frac{\sqrt{\epsilon_1^2 + \epsilon_2^2} - \sqrt{2(\epsilon_1 + \sqrt{\epsilon_1^2 + \epsilon_2^2})} + 1}{\sqrt{\epsilon_1^2 + \epsilon_2^2} + \sqrt{2(\epsilon_1 + \sqrt{\epsilon_1^2 + \epsilon_2^2})} + 1}. \tag{S2}$$

For the simulation of Tu *et al.*'s data at 300 K we used 13,600 cm^{-1} (5000 cm^{-1}), 170 cm^{-1} (2000 cm^{-1}) for ω_{p,D_1} (ω_{p,D_2}) and $1/\tau_{imp,1}$ ($1/\tau_{imp,2}$), respectively. For the simulation of our data we took 20,500 cm^{-1} (10,250 cm^{-1}), 128 cm^{-1} (2000 cm^{-1}) as ω_{p,D_1} (ω_{p,D_2}) and $1/\tau_{imp,1}$ ($1/\tau_{imp,2}$), respectively. For the two data we took the same four phonon modes: ($\omega_{p,l}$, ω_l, γ_l) = (400, 395, 15), (800, 482, 20), (800, 513, 28), and (570, 630, 25), respectively. We note that we simulate two cases with and without including the phonon modes to show the difference more clearly. For performing these simple simulations since we focus on data in a very low-frequency region ($\leq$ 600 cm^{-1}) we use only two Drude and four phonon modes excluding any other electronic contributions.

From the results of our simulations we can see clearly that the four phonon modes below 750 cm^{-1} are screened more strongly in our sample due to the enhanced Drude plasma frequency and reduced impurity scattering rate. Therefore, the phonon modes are suppressed significantly and appear quite weak in the simulated spectrum.

III. SIMULATION FORMALISM FOR OPTICAL CONSTANTS

We simulated some interesting optical quantities with the extracted electron-phonon spectral density function, $\alpha^2F(\omega)$, of our MgB_2 at 50 K using a reverse process[S5]. One may be interested in how much the characteristic energy scales of the optical quantities are dependent on the size of the superconducting gap. We simulated the optical conductivity data from the $\alpha^2F(\omega)$ at 50 K using the Allen's formula[S6] and an extended Drude model[S7,S8]. We calculate the imaginary part of the optical self-energy ($\Sigma_2^{op}(\omega)$) using the Allen's formula for

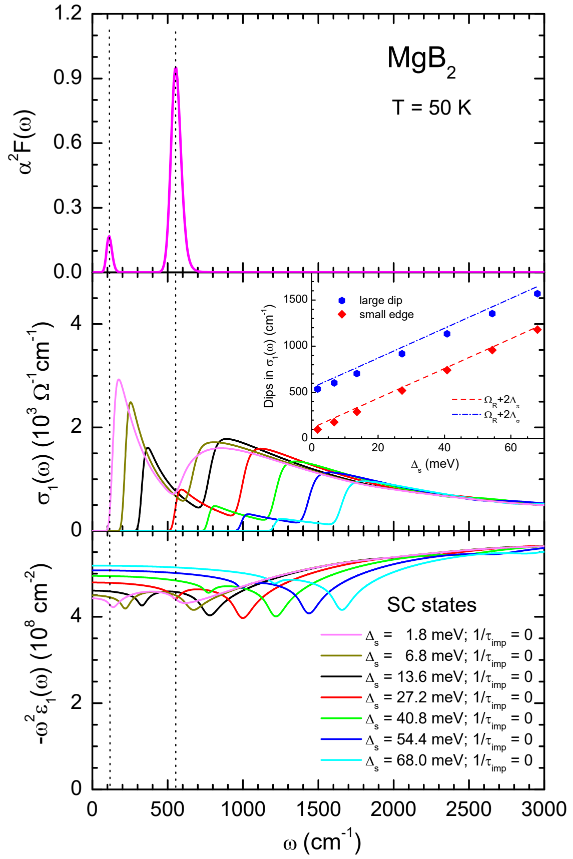

FIG. S3: (Top frame) We display the extracted $\alpha^2F(\omega)$ of MgB_2 at 50 K from the optical scattering rate using the Shulga *el al.*'s fromula[S3] and a maximum entropy method[S4]. (Middle frame) We display simulated real parts of the optical conductivity data in superconducting (SC) states with the extracted $\alpha^2F(\omega)$ and seven different sizes of the SC gaps using a so-called reverse process[S5] within the Allen's formalism for SC states[S6]. We assumed that the impurity scattering rate ($1/\tau_{imp}(\omega)$) is zero for all cases. In the inset we depict the dip position in $\sigma_1(\omega)$ as a function of Δ_s for the small peak (at $\Omega_R = 114$ cm^{-1}) and the large peak (at $\Omega_R = 550$ cm^{-1}). The straight lines show $\Omega_R + 2\Delta_s$ as a function of Δ_s for the two dips. (Bottom frame) We display $-\omega^2\epsilon_1(\omega)$ for the same cases as in the middle frame.

s-wave superconductors which we have already described in the main text as

$$-\Sigma_2^{op}(\omega) = \frac{\pi}{\omega}\int_0^{\omega-2\Delta_s}(\omega-\Omega)E\Big(\frac{\sqrt{(\omega-\Omega)^2-(2\Delta_s)^2}}{\omega-\Omega}\Big)d\Omega+\frac{1}{2\tau_{imp}}E\Big(\frac{\sqrt{\omega^2-(2\Delta_s)^2}}{\omega}\Big), \quad \text{(S3)}$$

where $\Sigma^{op}(\omega)_2$ is the imaginary part of the optical self-energy, which is related to the optical scattering rate as $\Sigma_2^{op}(\omega) = -1/2\tau^{op}(\omega)$, Δ_s is the superconducting gap, $1/\tau_{imp}$ is the impurity scattering rate, and $E(z)$ is the complete elliptic integral of the second kind. Once

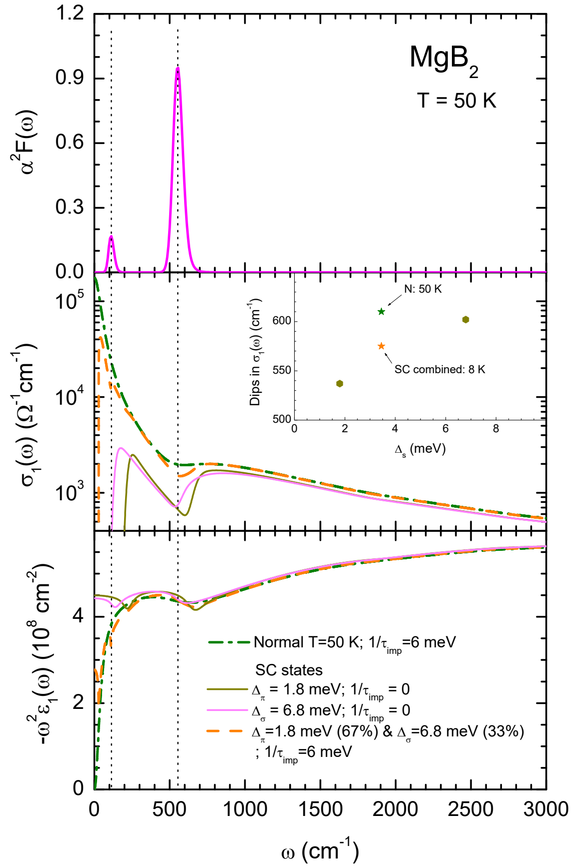

FIG. S4: (Top frame) We display the extracted $\alpha^2 F(\omega)$ of MgB_2 at 50 K from the optical scattering rate using the Shulga *el al.*'s fromula[S3] and a maximum entropy method[S4]. (Middle frame) We display simulated real parts of the optical conductivity data at 50 K (normal) with $1/\tau_{imp} = 6.0$ meV, in superconducting (SC) states with 2 different sizes of the SC gaps ($\Delta_s = 1.8$ and 6. 8 meV) with $1/\tau_{imp} = 0.0$ meV, and a combined case of the two SC gaps with $1/\tau_{imp} = 6.0$ meV using the extracted $\alpha^2 F(\omega)$ and the Allen's formula for SC states[S6]. In the inset we depict the dip position in $\sigma_1(\omega)$ as a function of Δ_s for the case of the large peak (at $\Omega_R = 550$ cm^{-1}): two separate SC gap (1.8 and 6.8 meV), the combined SC (8 K), and normal (50 K) cases. (Bottom frame) We display $-\omega^2\epsilon_1(\omega)$ for the same cases as in the middle frame.

one knows the imaginary part of the optical self-energy one can get its real part and then eventually can get the optical conductivity ($\tilde{\sigma}(\omega) = \sigma_1(\omega) + i\sigma_2(\omega)$) under the extended Drude formalism, which can be described as

$$\tilde{\sigma}(\omega) = \frac{\omega_p^2}{8\pi i}\frac{1}{\tilde{\Sigma}^{op}(\omega) - \omega/2} \ , \tag{S4}$$

where ω_p is the plasma frequency of charge carriers, which includes both contributions of coherent and incoherent components, and $\tilde{\Sigma}^{op}(\omega)$ $(= \Sigma_1^{op}(\omega) + i\Sigma_2^{op}(\omega))$ is the optical self-energy. The dielectric function $(\tilde{\epsilon}(\omega) = \epsilon_1(\omega) + i\epsilon_2(\omega))$ can be obtained using a formula, which relates it to the optical conductivity, as

$$\tilde{\epsilon}(\omega) - \epsilon_H = i\frac{4\pi}{\omega}\tilde{\sigma}(\omega)\ , \tag{S5}$$

where ϵ_H is a real number and the background dielectric constant which comes from contributions of the high energy responses, in principle, above the first interband transition. Furthermore, one also can calculate the reflectance out of the dielectric function using Eq. (S2).

In the top frame of Fig. S3 we display the extracted $\alpha^2F(\omega)$, which consists of two peaks: one is small and located at 114 cm^{-1} and the other is large and at 550 cm^{-1}. Using the reverse process which we described above we obtained the optical conductivity and the dielectric function for seven different sizes of the superconducting gaps (Δ_s = 1.8, 6.8, 13.6, 27.2, 40.8, 54.4, and 68 meV). In the middle frame of Fig. S3 we display the real part of the optical conductivity for the 7 cases. For each case we can see two characteristic energy scales: one is the conductivity edge which appears near Ω_R (= 114 cm^{-1}) + $2\Delta_s$ and the other is a dip near Ω_R (= 550 cm^{-1}) + $2\Delta_s$. In the inset we display the characteristic energy scales (symbols) as functions of SC gap (Δ_s) along with the corresponding $\Omega_R + 2\Delta_s$ (lines); the symbols are parallel to the lines. In the bottom frame of Fig. S3 we display $-\omega^2\epsilon_1(\omega)$ as a function of frequency. We can see the dips clearly near the characteristic energy scales in the optical conductivity. In general, the characteristic energy scales in the dielectric function are slightly blue-shifted compared with the corresponding energy scales in the conductivity. We note that when the peak energy (550 cm^{-1}) is very large compared with the size (1.8 meV $\cong$ 14.5 cm^{-1}) of the SC gap the dip in the dielectric function appears quite broad.

Now we compare the simulated data for MgB_2 at 50 K (normal) and 8 K (SC) using the extracted $\alpha^2F(\omega)$ and the reverse process[S5] and a similar approach[S9] which has been used to analyze the multiband correlated FeAs superconductors. For the normal case we obtained the imaginary part of the optical self-energy ($\Sigma_2^{op}(\omega)$) using the Shulga *et al.*'s formalism[S3],

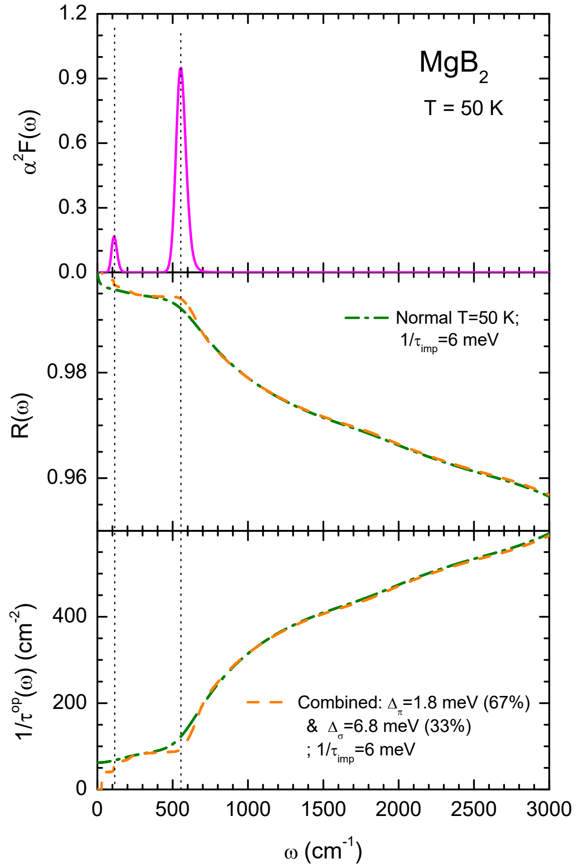

FIG. S5: (Top frame) We display the extracted $\alpha^2 F(\omega)$ of MgB_2 at 50 K from the optical scattering rate using the Shulga *el al.*'s fromula[S3] and a maximum entropy method[S4]. (Middle frame) We display simulated reflectance spectra at 50 K (normal) with $1/\tau_{imp}$ = 6.0 meV and a combined case of the two SC gaps ($\Delta_{s,\pi}$ = 1.8 meV and $\Delta_{s,\sigma}$ = 6.8 meV) using the extracted $\alpha^2 F(\omega)$ and the Allen's formula for SC states[S6]. (Bottom frame) We display $1/\tau^{op}(\omega)$ for the same cases of the middle frame.

which we have already descried in the main text as

$$-\Sigma_2^{op}(\omega, T) \;=\; \frac{\pi}{2\omega}\int_0^\infty \alpha^2 F(\Omega)\Big[2\omega \coth\Big(\frac{\Omega}{2T}\Big) - (\omega+\Omega)\coth\Big(\frac{\omega+\Omega}{2T}\Big) + \;(\omega-\Omega)\coth\Big(\frac{\omega-\Omega}{2T}\Big)\Big] d\Omega + \frac{1}{2\tau_{imp}}\;, \tag{S6}$$

where T is the absolute temperature and $1/\tau_{imp}$ is the impurity scattering rate. For the superconducting case the situation is more complicated since the data show the two SC gap channels distinctly. Therefore, we applied a method which has been used to analyze multi-band FeAs superconductors[S9]. The method is described briefly in the following sentences.

We calculated the optical conductivity ($\tilde{\sigma}(\omega)$) separately with 6.0 meV impurity scattering rate for each SC gap channel using the input $\alpha^2F(\omega)$ and Eq. (S3) through the reverse process[S5]. Then we obtained the combined optical conductivity of the two SC gap channels using the contribution portions which was suggested in a literature[S10]; 67% from the small SC gap (Δ_π) and 33% from the large SC gap (Δ_σ). Now we obtained the combined optical scattering rate from the combined optical conductivity using the extended Drude model. We also calculated the combined dielectric function from the conductivity using Eq. (S5) and eventually the combined reflectance from the dielectric function using Eq. (S2).

The top frame of Fig. S4 is the same as the one in the top frame of Fig. S3. In the middle frame of Fig. S4 two (dot-dashed olive and dashed orange) curves are the same curves in Fig. 4(b) of the main text. To compare with these two curves we show additional (solid purple and gold) curves which are brought from the middle frame of Fig. S3. The normal optical conductivity shows a quite broad kink (or dip) near 550 cm^{-1} where the large peak of $\alpha^2F(\omega)$ is located; since the small peak at 114 cm^{-1} is weak it is not easy to see the corresponding kink (or dip) clearly. The combined SC conductivity shows a clear dip around 575 cm^{-1}. The normal kink (or dip) is located around 610 cm^{-1}, which is slightly higher than the SC dip as shown in the inset where we display the positions of the normal kink (olive solid star), the SC dip (orange solid star) along with the corresponding dips (gold solid circle) for two SC gaps (1.8 meV and 6.8 meV). Actually, the 575 cm^{-1} SC dip has two components since it is obtained from the two parallel SC gap channels and, therefore, the position is located in between the dip energies of two separate SC gap cases. We note that the normal kink and SC dip appear at a similar energy scale with a small difference, 35 cm^{-1}. We display the corresponding $-\omega^2\epsilon_1(\omega)$ data in the bottom frame of Fig. S4.

Finally, in the middle and bottom frames of Fig. S5 we display the simulated reflectance spectra and optical scattering rates for normal (50 K) and SC (8 K) cases, respectively. We can see the characteristic energy scales quite clearly in both simulated reflectance spectra and optical scattering rates. We note that, in general, the optical scattering rate ($1/\tau^{op}(\omega)$) looks similar to $1 - R(\omega)$[S5].

[S1] Guritanu, V. *et al.* Anisotropic optical conductivity and two colors of MgB_2. *Phys. Rev. B* **73**, 104509 (2006).

[S2] Tu, J. J. *et al.* Optical properties of c-axis oriented superconducting MgB_2 films. *Phys. Rev. Lett.* **87**, 277001 (2001).

[S3] Shulga, S. V., Dolgov, O. V. & Maksimov, E. G. Electronic states and optical spectra of HTSC with electron-phonon coupling. *Physica C* **178**, 266 (1991).

[S4] Schachinger, E., Neuber, D. & Carbotte, J. P. Inversion techniques for optical conductivity data. *Phys. Rev. B* **73**, 184507 (2006).

[S5] Hwang, J. Reverse process of usual optical analysis of boson-exchange superconductors: impurity effects on *s*- and *d*-wave superconductors. *J. Phys.: Condens. Matter* **27**, 085701 (2015).

[S6] Allen, P. B. Electron-phonon effects in the infrared properties of metals. *Phys. Rev. B* **3**, 305 (1971).

[S7] Puchkov, A. V., Basov, D. N. & Timusk, T. The pseudogap state in high-T_c superconductors: an infrared study. *J. Phys.: Cond. Matter* **8**, 10049 (1996).

[S8] Hwang, J., Timusk, T. & Gu, G. D. High-transition-temperature superconductivity in the absence of the magnetic-resonance mode. *Nature (London)* **427**, 714 (2004).

[S9] Hwang, J. Electron-boson spectral density function of correlated multiband systems obtained from optical data: $Ba_0.6K_0.4Fe_2As_2$ and lifeas. *J. Phys.: Condens. Matter* **28**, 125702 (2016).

[S10] Dolgov, O. V. *et al.* Extraction of the electron-phonon interaction from tunneling data in the multigap superconductor MgB_2. *Phys. Rev. B* **68**, 132503 (2003).
